# Tumor necrosis factor‐α polymorphism and risk of primary nephrotic syndrome: A case–control study and meta‐analysis

**DOI:** 10.1002/iid3.1278

**Published:** 2024-06-11

**Authors:** Rongfen Zhao, Yufeng Wang, Yihua Zhou, Wu Fan, Haiyan Yin

**Affiliations:** ^1^ Department of Clinical Laboratory The People's Hospital of Suzhou New District Suzhou Jiangsu China

**Keywords:** gene polymorphism, meta‐analysis, novel marker, primary nephrotic syndrome, TNF‐α

## Abstract

**Background:**

The current study aims to explore the relationship between tumor necrosis factor‐α (TNF‐α) polymorphism and the risk of primary nephrotic syndrome (PNS).

**Methods:**

A total of 250 PNS patients were selected for this study, as well as 300 volunteers serving as the control group. TNF‐α polymorphism were assessed using the polymerase chain reaction‐restriction fragment length polymorphism method. In addition, a meta‐analysis was conducted to analyze previously published literature on this topic.

**Results:**

No significant differences were observed in the genotypes frequency or alleles frequency among the study populations. Meta‐analysis results revealed a positive association between TNF‐α rs1800629 polymorphism and allele contrast in African populations (*p* = 0), homozygote comparison (*p* = .007), heterozygote comparison (*p* = .026), recessive genetic model (*p* = .011), and dominant genetic model (*p* = .000).

**Conclusions:**

TNF‐α rs1800629 polymorphism does not appear to confer any increased risk for PNS.

## 1 INTRODUCTION

Primary nephrotic syndrome (PNS) is a clinical syndrome characterized by the excessive loss of plasma proteins in urine due to increased glomerular basement membrane permeability. The etiology of PNS is not yet fully understood, and its occurrence and development are believed to be influenced by various factors, including heredity, environment, humoral factors, and immune responses.^1^ PNS primarily involves immune‐mediated glomerular injury, with minimal change disease (MCD), focal segmental sclerosis (FSGS), membranous nephropathy (MN), and mesangial proliferative glomerulonephritis (MsPGN) being the main pathological types observed in patients.^2^, ^3^ These immune‐mediated autoimmune diseases significantly impact human health.

Tumor necrosis factor‐α (TNF‐α) is a crucial regulator of inflammation and immune responses with multidirectional effects.^4^, ^5^, ^6^, ^7^ It is closely associated with glomerular injury and has been extensively studied in multiple diseases, including malignancies,^8^, ^9^, ^10^ inflammatory diseases,^11^, ^12^ autoimmune diseases,^13^, ^14^ cardiovascular and cerebrovascular diseases,^15^ as well as infectious diseases.^12^, ^16^ The TNF‐α gene polymorphism is known to influence gene transcription and cytokine synthesis. Among these polymorphisms, TNF‐α rs1800629 polymorphism has been extensively investigated in various disorders. Furthermore, the association between TNF‐alpha gene polymorphisms and childhood nephrotic syndrome has been explored.^17^ This particular SNP has been found to have a significant impact on gene transcription and cytokine synthesis within multiple disease contexts.^18^

Despite comprehensive investigations on TNF‐α polymorphisms, including rs1800629, the specific impact of TNF‐α rs1800629 polymorphism on the risk of developing PNS remains to be elucidated.

The aim of this study is to contribute to our understanding of the etiology and risk factors associated with PNS by exploring the potential relationship between TNF‐α rs1800629 polymorphism and PNS risk using experimental method and meta‐analysis. The findings from this research may have significant implications for the prevention and management of PNS and provide valuable insights for future clinical interventions.

## 2 MATERIALS AND METHODS

### 2.1 Materials

#### 2.1.1 Chemicals and reagents

A list of the main chemicals and reagents used in our research, together with their respective manufacturers, suppliers and product details, is given in Supporting Information.

### 2.2 Participates characteristics

A total of 250 patients diagnosed with PNS were recruited from The People's Hospital of Suzhou New District between March 2018 and March 2023. All patients met the diagnostic criteria of PNS, which included a 24‐h urinary protein level greater than 3.5 g/day and serum protein level below 30 g/L. The patients underwent renal biopsy and had not received hormone drugs within 2 weeks before admission. Exclusion criteria were defined as follows: (1) subsequent nephrotic syndrome, (2) co‐infection with fungal, bacterial, or mycoplasma infections, (3) presence of malignant tumors including blood diseases and solid tumors, (4) history of tissue or organ injury such as recent trauma, burn, surgery, or myocardial infarction, and (5) presence of autoimmune diseases such as systemic lupus erythematosus or rheumatoid arthritis.

The control group consisted of 300 healthy volunteers who underwent physical examination during the same period. Control participants had normal blood routine, urine routine, liver function, kidney function and did not have glomerular diseases, tumors or other diseases. Informed consent was obtained from all participants. Detailed demographic characteristics such as age, sex, body mass index (BMI), smoking status, and alcohol consumption were recorded for both PNS patients and control participants (Table 1).

**Table 1:** The participates characteristics of both PNS group and control group.

Basic information		Control (*N* = 300)	PNS (*N* = 250)	*p*
Age		38.4 ± 8.9	36.9 ± 11.3	.28
Sex	Male	182	160	.42
	Female	118	90	
Smoking	Yes	155	138	.41
	No	145	112	
Alcohol consumption	Yes	98	88	.53
	No	202	162	
BMI	BMI < 18.5 kg//m^2^	65	40	.09
	BMI ≥ 18.5 kg//m^2^	235	210	

Abbreviations: BMI, body mass index; PNS, primary nephrotic syndrome.

### 2.3 TNF‐α polymorphism analysis

Blood samples obtained from all participants were processed according to standard procedures using a DNA extraction kit. The TNF‐α rs1800629 polymorphism was determined using the polymerase chain reaction‐restriction fragment length polymorphism (PCR‐RFLP) method, which has been extensively described in previous studies. DNA sequencing was performed to validate the genotyping results for a total of 550 samples. The primer sequences used for the TNF‐α rs1800629 polymorphism analysis were as follows: forward: 5′‐GGAGGCAATAGGTTTTGAGGGCCAT‐3′; reverse: 5′‐CTGCACCTTCTGTCTCGGTTTCT‐3′.

### 2.4 The process of meta‐analysis

A comprehensive literature search was conducted in well‐established databases, including PubMed and EMBASE. The search covered articles published from the inception of these databases until December 2022. Search terms included “polymorphism” or “polymorphisms” together with “primary nephrotic syndrome” or “PNS” and “tumor necrosis factor‐alpha” or “TNF‐alpha.” Only studies that met the following criteria were included in the meta‐analysis: (a) case–control studies investigating the association of interest, and (b) studies providing data to estimate effect sizes. Studies were excluded if they met any of the following criteria: (a) not being a case‐control study involving human subjects, or (b) lacking sufficient data to estimate effect sizes. Two authors independently conducted the search, review, and evaluation of all included studies. Relevant information such as author names, publication year, and Hardy‐Weinberg equilibrium (HWE) status were recorded. The Newcastle–Ottawa Scale (NOS) score was used to assess study quality based on these criteria.^18^

### 2.5 Statistical analysis

Statistical analysis was performed using SPSS version 17.0 software. Measurement data were presented as means ± standard deviation (SD), and comparisons between independent samples were performed using *t*‐tests. Categorical data were analyzed using chi‐square tests. The meta‐analysis was carried out based on the selected literature sources.^19^, ^20^, ^21^, ^22^, ^23^, ^24^

## 3 RESULTS

### 3.1 Participants enrolled in the study

Table 1 described the participants' information of PNS patients and healthy controls. No significant difference was found in the above information which was referred in Section 2 (*p* > .05).

### 3.2 TNF‐α polymorphism

A total of three genotypes (GA, AA, and GG) were found in the present experiment. In the present experiment, we found no noteworthy difference of distribution rate in both allele and genotype. The detailed information was shown in Table 2. The distribution frequency of TNF‐α rs1800629 genotype in PNS group showed no significant bias in gender and age.

**Table 2:** Comparison of genotype and allele frequency between PNS group and control group.

TNF‐α rs1800629	Control group (*N* = 300)		PNS group (*N* = 250)		OR (95%CI)^a^	*p* a
	*n*	Percentage (%)	*n*	Percentage (%)		
GA	84	28.0	72	28.8	1.00^REF^	
AA	98	32.7	78	31.2	0.93 (0.60–1.43)	.737
GG	118	39.3	100	40.0	0.99 (0.65–1.49)	.957
G	320	53.3	272	54.4	1.00^REF^	
A	280	46.7	228	45.6	1.00 (0.79–1.27)	.982

Abbreviations: CI, confidence interval; PNS, primary nephrotic syndrome; OR, odds ratio.

^a^ Adjusted for sex and age by logistic regression model.

### 3.3 Meta‐analysis results

**Figure 1:**
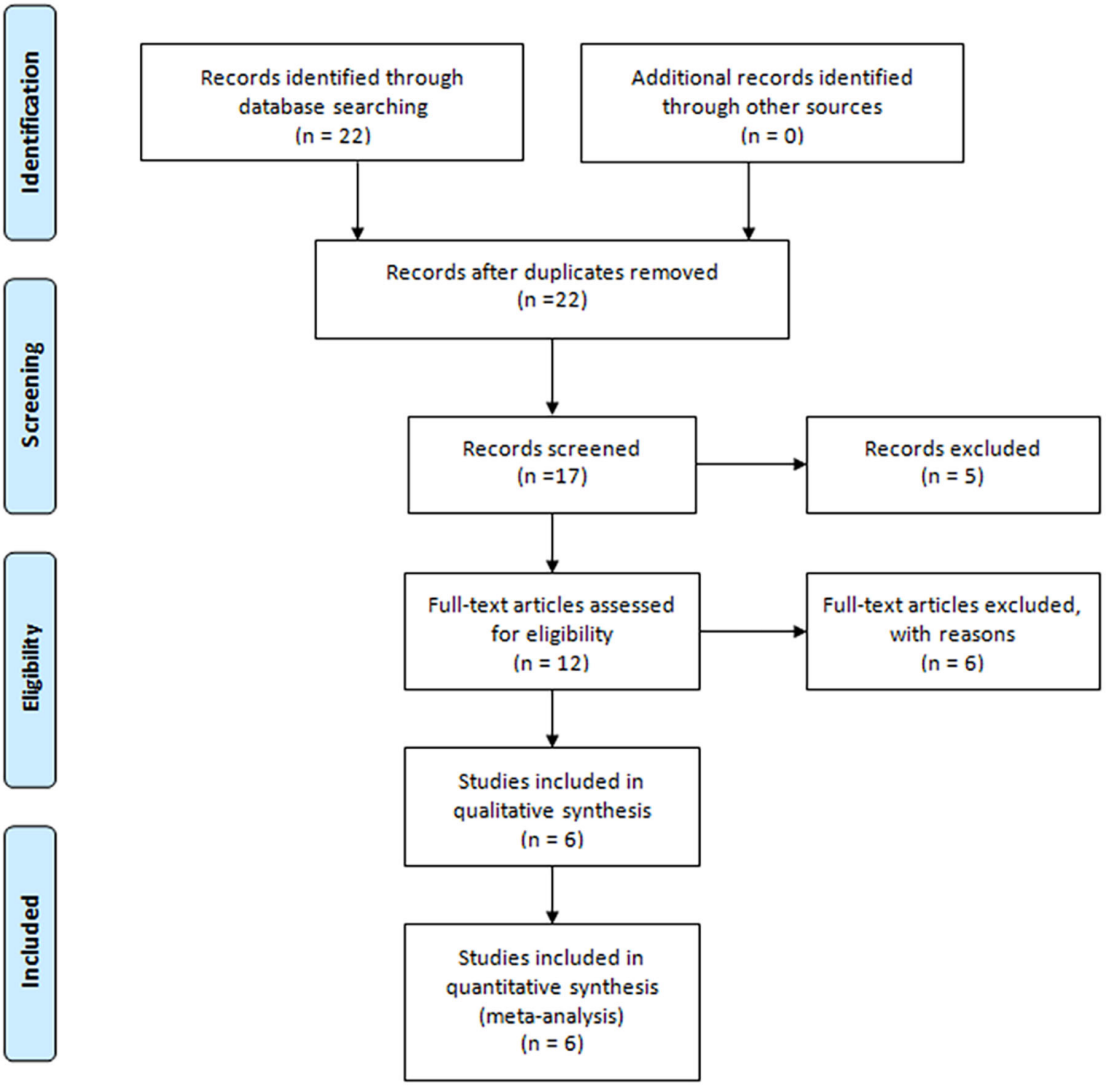
PRISMA 2009 flow diagram.

**Figure 2:**
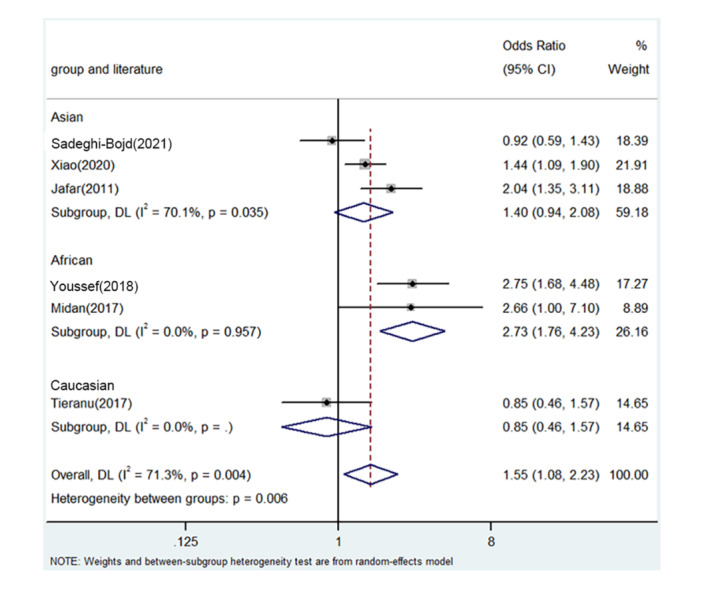
Forest plot for the associations between TNF‐α rs1800629 polymorphism and PNS risk through allele contrast (A vs. G). CI, confidence interval; OR, odds ratio; PNS, primary nephrotic syndrome; TNF‐α, tumor necrosis factor‐α.

**Figure 3:**
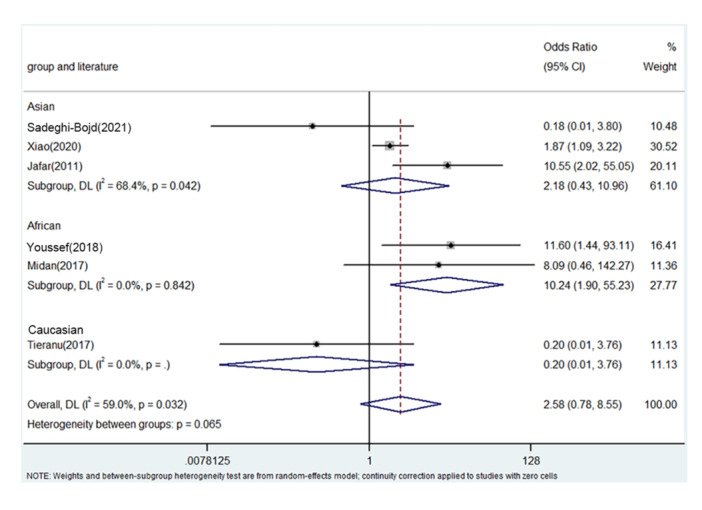
Forest plot for the associations between TNF‐α rs1800629 polymorphism and PNS risk through homozygote comparison (AA vs. GG). CI, confidence interval; OR, odds ratio; PNS, primary nephrotic syndrome; TNF‐α, tumor necrosis factor‐α.

**Figure 4:**
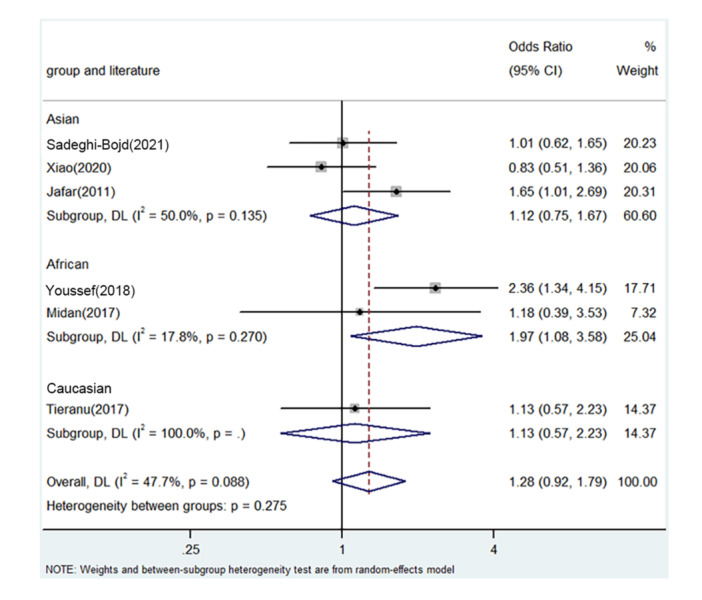
Forest plot for the associations between TNF‐α rs1800629 polymorphism and PNS risk through heterozygosis comparison (GA vs. GG). CI, confidence interval; OR, odds ratio; PNS, primary nephrotic syndrome; TNF‐α, tumor necrosis factor‐α.

**Figure 5:**
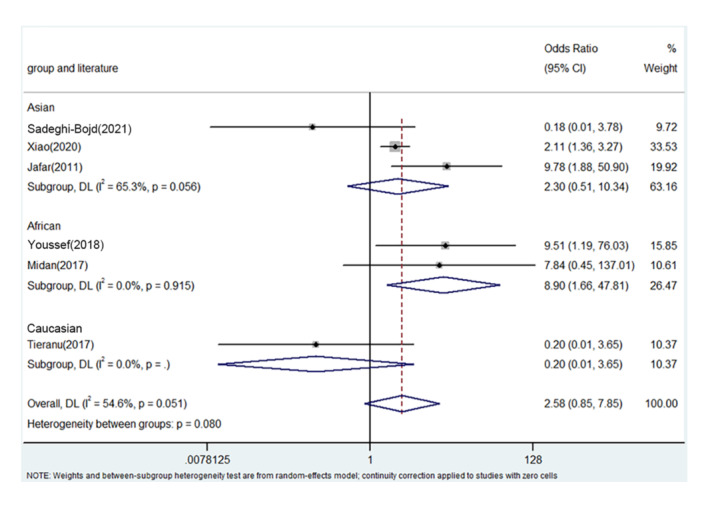
Forest plot for the associations between TNF‐α rs1800629 polymorphism and PNS risk through recessive genetic model (AA vs. AG/GG). CI, confidence interval; OR, odds ratio; PNS, primary nephrotic syndrome; TNF‐α, tumor necrosis factor‐α.

**Figure 6:**
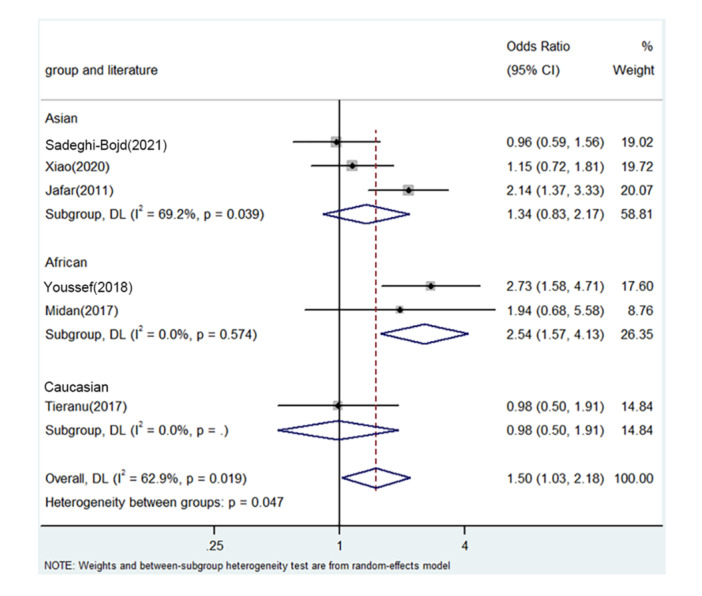
Forest plot for the associations between TNF‐α rs1800629 polymorphism and PNS risk through dominate genetic model (AA/GA vs. GG). CI, confidence interval; OR, odds ratio; PNS, primary nephrotic syndrome; TNF‐α, tumor necrosis factor‐α.

PRISMA 2009 Flow Diagram (Figure 1) described the whole process of searching. Six literatures from five countries were enrolled including China, Egypt, Iran, Romania, and India.^18^, ^25^, ^26^, ^27^, ^28^, ^29^ Table 3 describes the whole data and information. Table 4 describes the whole NOS results. The positive findings were shown in African population by allele contrast (*p* = 0, Figure 2), homozygote comparison (*p* = .007, Figure 3), heterozygote comparison (*p* = .026, Figure 4), recessive genetic model (*p* = .011, Figure 5), and dominate genetic model (*p* = 0, Figure 6). The main results were shown in Table 5.

**Table 3:** Main characteristics of all case‐control studies included in meta‐analysis.

Literature	Ethnics (Country)	Genotyping methods	Source of control	Sample size	HWE conformity	NOS	Genotype frequency (Case)			Genotype frequency (Control)			Year
							GG	GA	AA	GG	GA	AA	
Sadeghi‐Bojd et al.^18^	Asian (Iran)	PCR‐RFLP	PB	168/153	Yes	8	121	47	0	109	42	2	2021
Xiao et al.^25^	Asian (China)	DNA sequence	PB	200/200	Yes	8	46	78	76	51	104	45	2020
Youssef et al.^26^	African (Egypt)	PCR‐RFLP	HB	150/150	Yes	9	97	44	9	125	24	1	2017
Midan et al.^27^	African (Egypt)	PCR‐RFLP	HB	100/30	Yes	8	72	17	11	25	5	0	2017
Tieranu et al.^28^	Caucasian(Romania)	TaqMan	PB	70/159	Yes	8	54	16	0	122	32	5	2017
Jafar et al.^29^	Asian (India)	PCR‐RFLP	PB	150/569	Yes	9	118	27	5	498	69	2	2011

Abbreviations: HB, hospital‐based; HWE, Hardy–Weinberg equilibrium; RFLP, restricted fragment length polymorphism; NOS, Newcastle–Ottawa Score; PB, population‐based.

**Table 4:** Quality assessment of the six case–control studies according to the Newcastle–Ottawa scale.

Literature	Selection of enrolled study subjects	Between‐group comparability	Exposure outcomes and factors	Total
Sadeghi‐Bojd et al.^18^	3	2	3	8
Xiao et al.^25^	3	2	3	8
Youssef et al.^26^	4	3	2	9
Midan et al.^27^	3	2	3	8
Tieranu et al.^28^	3	2	3	8
Jafar et al.^29^	4	3	2	9
Average	3.1	2.3	2.7	8.1

**Table 5:** Meta‐analysis of the TNF‐α rs1800629 polymorphism and PNS risk.

Comparison	Population	*N*	Test of association			Mode	Test of heterogeneity		
			OR	95%CI	*p*		*χ* ^2^	*p*	*I* ^2^
A versus. G	Overall	6	1.55	1.08–2.23	.019	Random	19.47	.004	71.3
	Asian	3	1.40	0.94–2.08	.094	Random	5.21	.035	70.1
	African	2	2.73	1.76–4.23	0	fixed	0.03	.957	0
	Caucasian	1	1.55	1.08–2.23	.598	/	/	/	/
AA versus. GG	Overall	6	2.58	0.78–8.55	.121	Random	12.28	.032	59.0
	Asian	3	2.18	0.43–10.96	.344	Random	3.72	.042	68.4
	African	2	10.24	1.90–55.23	.007	fixed	0.81	.842	0
	Caucasian	1	0.20	0.01–3.76	.285	/	/	/	/
GA versus. GG	Overall	6	1.28	0.92–1.79	.143	Random	4.00	.088	47.7
	Asian	3	1.12	0.75–1.67	.591	Random	4.00	.135	50.0
	African	2	1.97	1.08–3.58	.026	fixed	1.22	.270	17.8
	Caucasian	1	1.13	0.57–2.23	.725	/	/	/	/
AA versus. GA/GG	Overall	6	2.58	0.85–7.85	.096	Random	11.05	.051	54.6
	Asian	3	2.30	0.51–10.34	.278	Random	3.05	.056	65.3
	African	2	8.90	1.66–47.81	.011	fixed	0.76	.915	0
	Caucasian	1	0.20	0.01–3.65	.277	/	/	/	/
AA/GA versus. GG	Overall	6	1.50	1.03–2.18	.036	Random	14.48	.019	62.9
	Asian	3	1.34	0.83–2.17	.232	Random	7.00	.039	69.2
	African	2	2.54	1.57–4.13	0	fixed	0.11	.574	0
	Caucasian	1	0.98	0.50–1.91	.946	/	/	/	/

Abbreviations: CI, confidence interval; PNS, Primary nephrotic syndrome; OR, odds ratio; TNF‐α, tumor necrosis factor‐α.

## 4 DISCUSSION

Our study aimed to investigate the association between TNF‐α rs1800629 polymorphism and the risk of primary nephrotic syndrome (PNS). However, we did not find a significant association between TNF‐α rs1800629 polymorphism and PNS risk in our study. Neither genotype nor allele frequencies demonstrated a significant influence on the risk of PNS. This finding suggests that this particular polymorphism may not contribute to an increased or decreased risk of developing PNS.

Interestingly, our findings contrast with some earlier studies. Jafar et al. concluded that TNF‐α rs1800629 polymorphism may affect susceptibility to idiopathic nephrotic syndrome,^29^ and Midan et al.^27^ also proposed that this polymorphism might influence the risk of idiopathic nephrotic syndrome. These discrepancies are not uncommon in genetic association studies and can be attributed to various factors, including differences in the study population, racial backgrounds, sample sizes, and other confounding variables. In our study, the population characteristics and other variables were different from those of studies conducted in India and Iran. Although we obtain a positive result by meta‐analysis, we believe that the positive results are due to factors such as racial differences and geographical differences. There is much evidence for this in the previously reported literature. Therefore, it is not uncommon for the results of meta‐analyses to be inconsistent with those of individual case‐control studies.

When considering our findings in the context of immunity, inflammation, and disease progression, it is important to note that TNF‐α is a key cytokine involved in both immune responses and inflammation.^30^ It plays a crucial role in regulating immune cell activation, cytokine production, cell proliferation, apoptosis, and tissue repair processes.^31^ In PNS, immune‐mediated glomerular injury is considered a major pathogenic mechanism.^32^ The balance between pro‐inflammatory cytokines, such as TNF‐α, and anti‐inflammatory cytokines is crucial for maintaining immune homeostasis and preventing excessive inflammation. Disruptions in this balance can contribute to the development and progression of various autoimmune diseases, including PNS.

While our study did not reveal a direct association between TNF‐α rs1800629 polymorphism and PNS risk, it is possible that this polymorphism may have an indirect impact on disease progression and severity through its effects on urinary protein levels. Microalbuminuria, considered an early marker of kidney damage, has been associated with increased risk of CKD progression.^33^

This study holds several strengths and limitations. Firstly, this study is the first to investigate TNF‐α rs1800629 polymorphism in PNS patients both domestically and internationally, contributing to the existing literature on this topic. Additionally, the sample size of this study is relatively large, with more than 500 individuals enrolled in the analysis. The inclusion of a substantial sample size is crucial for the study of gene polymorphism and has been demonstrated to be beneficial in previous literature.^34^, ^35^, ^36^, ^37^, ^38^, ^39^, ^40^ However, there are certain limitations that must be acknowledged. Although our study design was innovative in exploring TNF‐α rs1800629 polymorphism in PNS patients, there is always room for improvement. Future research should consider incorporating bioinformatics techniques, which can enhance diagnostic accuracy, prognostic evaluation, and treatment strategies for various diseases.^41^, ^42^, ^43^, ^44^ Furthermore, well‐designed multicenter studies with larger sample sizes are needed to further explore this association using methods such as genome‐wide association studies and updated meta‐analytical approaches.

## 5 CONCLUSION

Our results indicated that TNF‐α rs1800629 polymorphism conferred no risk to PNS.

## AUTHOR CONTRIBUTIONS

**Rongfen Zhao**: Conceptualization; data curation; writing—original draft. **Yufeng Wang**: Formal analysis; investigation; methodology; software. **Yihua Zhou**: Data curation; formal analysis; investigation. **Wu Fan**: Data curation; investigation. **Haiyan Yin**: Conceptualization; formal analysis; writing—original draft; writing—review & editing.

## CONFLICT OF INTEREST STATEMENT

The authors declare no conflict of interest.

## ETHICS STATEMENT

This study was conducted with approval from the Ethics Committee of our hospital [No. L2023229]. This study was conducted in accordance with the declaration of Helsinki. Written informed consent was obtained from all participants.

## Supporting information

Supporting information.

## Data Availability

The data that support the findings of this study are available from the corresponding author, upon reasonable request. All data generated or analyzed during this study are included in this published article.
