# Supplementary material for: Tumor necrosis factor‐α polymorphism and risk of primary nephrotic syndrome: A case–control study and meta‐analysis
Source: Immun Inflamm Dis. 2024 Jun 11;12(6):e1278. doi: 10.1002/iid3.1278 (PMC11165685; doi:10.1002/iid3.1278)
Supplement: Supplementary file 1 — Supporting information. [file IID3-12-e1278-s001.docx]

Chemicals and Reagents

1.DNA polymerase: Purchased from Takara Biotechnology (Dalian) Co., Ltd.

2.dNTPs: Purchased from Takara Biotechnology (Dalian) Co., Ltd.

3.10*PCR buffer: Purchased from Takara Biotechnology (Dalian) Co., Ltd.

4.5*Loading buffer: Purchased from Takara Biotechnology (Dalian) Co., Ltd.

5.DNA marker: Purchased from Jingmei Biotechnology Co., Ltd.

6.DNA extraction kit: Purchased from Qiagen Co., Ltd.

7.Primer: Synthesized by ShangHai RealGene Biotech, Inc.

Instruments

1.Microcentrifuge tube: Manufactured by Jingmei Biotechnology Co., Ltd.

2.Electric thermostatic water bath: Manufactured by Shanghai medical constant temperature equipment factory.

3.Optical microscope: Manufactured by Olympus Corporation, Japan.

4.Spectrophotometer: Manufactured by Backman Coulter, USA.

5.Fully automatic enzyme marker: Labsystem Wellscan MK.
